# Porphyrin Homeostasis Maintained by ABCG2 Regulates Self-Renewal of Embryonic Stem Cells

**DOI:** 10.1371/journal.pone.0004023

**Published:** 2008-12-24

**Authors:** Jimmy Susanto, Yu-Hsing Lin, Yun-Nan Chen, Chia-Rui Shen, Yu-Ting Yan, Sheng-Ta Tsai, Chung-Hsuan Chen, Chia-Ning Shen

**Affiliations:** 1 Genomics Research Center, Academia Sinica, Taipei, Taiwan, Republic of China; 2 Graduate Institute of Life Sciences, National Defense Medical Center, Taipei, Taiwan, Republic of China; 3 Department of Biotechnology and Laboratory Science in Medicine, National Yang-Ming University, Taipei, Taiwan, Republic of China; 4 Department of Medical Biotechnology and Laboratory Science, Chang Gung University, Tao-Yuan, Taiwan, Republic of China; 5 Institute of Biomedical Science, Academia Sinica, Taipei, Taiwan, Republic of China; Ordway Research Institute, United States of America

## Abstract

**Background:**

Under appropriate culture conditions, undifferentiated embryonic stem (ES) cells can undergo multiple self-renewal cycles without loss of pluripotency suggesting they must be equipped with specific defense mechanisms to ensure sufficient genetic stability during self-renewal expansion. The ATP binding cassette transporter ABCG2 is expressed in a wide variety of somatic and embryonic stem cells. However, whether it plays an important role in stem cell maintenance remains to be defined.

**Methodology/Principal Findings:**

Here we provide evidence to show that an increase in the level of ABCG2 was observed accompanied by ES colony expansion and then were followed by decreases in the level of protoporphyrin IX (PPIX) indicating that ABCG2 plays a role in maintaining porphyrin homoeostasis. RNA-interference mediated inhibition of ABCG2 as well as functional blockage of ABCG2 transporter with fumitremorgin C (FTC), a specific and potent inhibitor of ABCG2, not only elevated the cellular level of PPIX, but also arrest the cell cycle and reduced expression of the pluripotent gene *Nanog*. Overexpression of ABCG2 in ES cells was able to counteract the increase of endogenous PPIX induced by treatment with 5-Aminolevulinic acid suggesting ABCG2 played a direct role in removal of PPIX from ES cells. We also found that excess PPIX in ES cells led to elevated levels of reactive oxygen species which in turn triggered DNA damage signals as indicated by increased levels of γH2AX and phosphorylated p53. The increased level of p53 reduced Nanog expression because RNA- interference mediated inhibition of p53 was able to prevent the downregulation of Nanog induced by FTC treatment.

**Conclusions/Significance:**

The present work demonstrated that ABCG2 protects ES cells from PPIX accumulation during colony expansion, and that p53 and γH2AX acts as a downstream checkpoint of ABCG2-dependent defense machinery in order to maintain the self-renewal of ES cells.

## Introduction

Embryonic stem (ES) cells are pluripotent cells derived from the inner cell mass of blastocysts. Under appropriate culture conditions, undifferentiated ES cells can be maintained over many self-renewal cycles without loss of pluripotency [1], [2]. Moreover, ES cells are unique in that unlike differentiated cells they do not accumulate DNA damage during multiple self-renewal cycles. This feature is important *in vivo* as a small number of ES cells can contribute the whole process of embryogenesis, therefore if DNA damage accumulated in ES cells could potentially affect development of different tissue types.

One of the major causes of DNA damage in cells is reactive oxygen species (ROS). Several studies have shown that low/moderate levels of ROS generated from cell metabolism play an important role in maintenance physiological functions of cells and in some cases are even used as the signaling mediator [3]. However, high levels of ROS may cause damages to cell structures, including lipids and membranes, proteins, and DNA, which can in turn lead to apoptosis or senescence [4]. In fact, it has been shown that the mutation frequency in ES cells is low because ES cells are sensitive to DNA damage and readily undergo apoptosis or differentiation in order to remove damaged cells from the self-renewal pool [5]–[7]. Moreover, in order to prevent excessive ROS levels ES cells express high levels of antioxidant defense enzymes as well as high activity of verapamil-sensitive multidrug transporter [8], [9].

The ATP binding cassette transporter ABCG2 is a verapamil-sensitive multidrug transporter that is expressed in a wide variety of drug-resistant cancer cells, extrudes xenobiotics and certain drugs from cells, thereby mediating drug resistance and affecting the pharmacological behavior of many compounds [10]–[12]. Later studies determined that ABCG2 expression is not unique to drug resistant cancer cells, but is also expressed in a wide variety of stem cells and in numerous adult tissues [1], [2]. In fact, ABCG2 is also the molecular determinant of the side-population (SP) phenotype, which has been widely used for the detection and enrichment of tissue stem cells [1], [10]. ABCG2 was also found to be highly expressed in human ES cells [13] as well as rhesus monkey ES cells [14]. Interestingly despite the clear correlation between ABCG2 and stem cells, its exact function in these cells has not been elucidated.

Recently it has been shown that ABCG2 plays a role in enhancing the survival of haematopoetic stem cells in hypoxia, which is possibly mediated through transportation of heme and porphyrins [15]. Heme is composed of iron and protoporphyrin IX (PPIX) which is s an essential component of various hemoproteins, including cytochromes involved in mitochondrial electron transfer chain and in drug metabolism [16]. Hemes are also important cofactors in oxygen storage and transport (such as hemoglobin and myoglobin), signaling mediator (nitric oxide synthases, guanylate cyclases) and in regulation of antioxidant-defense enzymes [16], [17]. The levels of PPIX in cells are tightly regulated in many cell types as excess PPIX could undergo the iron catalyzed fenton reaction and generate potentially DNA damaging ROS [16]. Recently identified heme/porphyrin transporters such as heme carrier protein 1 (HCP1), FLVCR, ABCB6 and ABCG2 are expected to play an important role in maintaining a homeostatic level of porphyrins

Developing embryos naturally resides in hypoxic microenvironments and low level of oxygen regulates cell fate decision of embryonic stem/progenitor cells. Recent work further suggests undifferentiated mouse ES cells adapt their energy metabolism to proliferate at different oxygen tension [18]. Cellular adaptations to changes in oxygen levels include stimulating several hypoxia-inducible factors that mediate oxygen homeostasis and control the level of heme, a molecule whose level changes in response to changes in cellular oxygen [19], [20]. ABCG2 expression is upregulated under low oxygen conditions, which is consistent with its high expression in tissues exposed to low oxygen environments [21]. Since ABCG2 interacts with porphyrin [15], [21], [22] and elevated levels of PPIX in erythroid progenitors of ABCG2-deficient mice support the idea that porphyrins are endogenous ABCG2 substrates [23]. We speculate PPIX homeostasis in ES cells is maintained by ABCG2 in order to adapt to changes in oxygen availability during rapid colony expansion. However, if ABCG2 function was disrupted, the accumulation of PPIX possibly lead to increased level of ROS would therefore induce DNA damage and then trigger downstream checkpoint signals, which may in turn cause the ES cell to lose its pluripotency as well as disrupting the self-renewal cycle.

## Results

### Inhibition of ABCG2 leads to downregulation of Nanog in mouse ES cells

**Figure 1:**
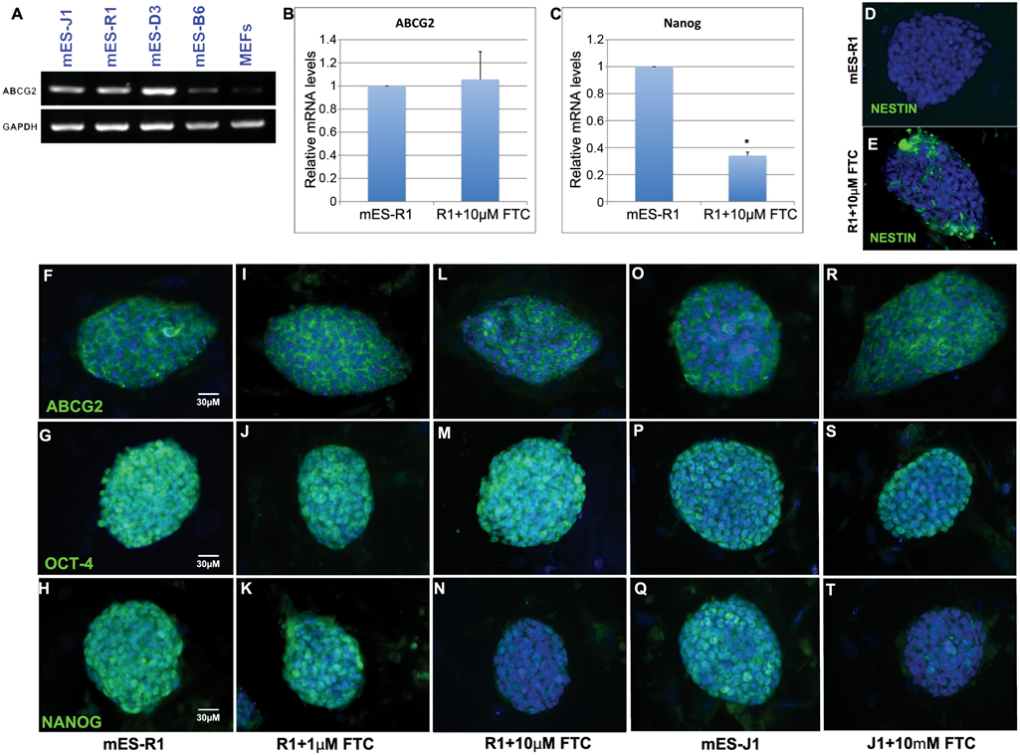
Inhibition of ABCG2 led to downregulation of Nanog in ES cells. (A) RT-PCR data of ABCG2 in J1, R1, D3 and B6 mouse ES cell lines as well as in MEFs, with GAPDH as internal control. (B, C) Real-time PCR data showing relative mRNA expression level of ABCG2 (B) and Nanog (C) in mES-R1 cells treated with or without 10 µM of the ABCG2 inhibitor FTC, normalized to GAPDH. Data was calculated from at least three independent experiments. A t-test was performed to confirm statistical significance and * indicates p<0.05 (D, E) Immunofluorescence staining showing Nestin expression was observed in FTC treated mES-R1 cells. (F–T) Immunofluorescence staining for ABCG2, Oct-4 and Nanog in untreated mES-R1 and mES-J1 cells, in mES-R1 cells treated with 1 µM or 10 µM of the ABCG2 inhibitor FTC, and in mES-J1 cells treated with 10 µM FTC for 72 hours.

No detailed data was available regarding the expression of ABCG2 in mouse ES cells. Therefore, we first set out to determine whether ABCG2 is expressed in mouse ES cells. Using RT-PCR, we found that ABCG2 is expressed in several mouse ES cell lines, including ES-R1, J1, D3, and B6 (Fig. 1A). The expression levels varied among the cell lines, with the highest level seen in ES-D3 cells and the lowest level observed in ES-B6 cells.

After establishing the expression pattern of ABCG2 in mouse ES cells, we next investigated its possible function. To do this, we utilized the chemical inhibitor fumitremorgin C (FTC), which specifically inhibits the activity of ABCG2 [24]. Verapamil was not used as it can also inhibit other multidrug transporter [25], [26]. When mouse ES cells were treated with either 1 or 10 µM FTC for 72 hours, we observed that expression levels of ABCG2 remained similar to those of control cells (Fig 1B). However, Nanog expression was significantly downregulated in the 10 µM FTC-treated cells, as observed by both Immunofluorescence staining and real-time RT-PCR (Fig. 1C, F–T). We were also able to detect expression of the early ectodermal marker Nestin in FTC treated mES cells indicating perhaps that some of the ES cells have started to undergo differentiation.

**Figure 2:**
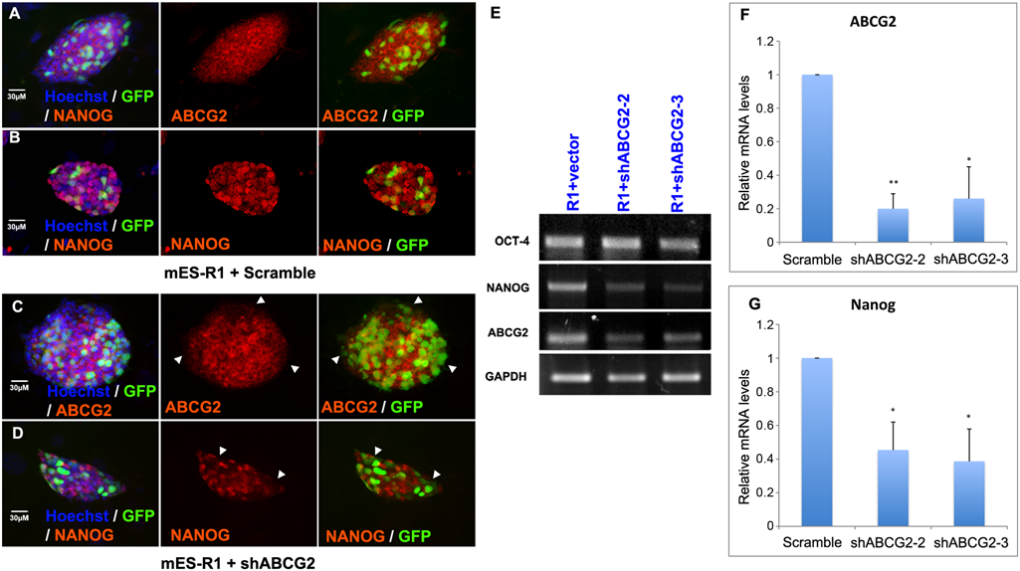
Knock-down of ABCG2 led to downregulation of Nanog. (A–D) Immunofluorescence staining for mES-R1 cells transfected with control scramble construct (A, B) and shABCG2-3 construct (C, D) and. The GFP marked transfected cells and red represented ABCG2 (A, C) and Nanog (B, D) staining. Immunofluorescence staining was performed at 72 hours after transfection. Images represent transfections with either shABCG2-2 or shABCG2-3. (E) RT-PCR data for Oct-4, Nanog and ABCG2 for mES-R1 cells transfected with scrambled, shABCG2-2 or shABCG2-3 with GAPDH as internal control. (F, G) Real-time PCR data showing relative mRNA expression level of ABCG2 (F) and Nanog (G) in mES-R1 cells transfected with scrambled, shABCG2-2 or shABCG2-3 normalized to GAPDH. A t-test was performed to confirm statistical significance and * indicates p<0.05 and ** indicates p<0.01. Data was calculated from at least three independent experiments.

To confirm whether the downregulation of Nanog was specifically caused by inhibition of ABCG2, we designed two shRNA constructs based on RNAi Codex database [27]: shABCG2-2 and shABCG2-3, which specifically target the 5′ and 3′ ends of ABCG2. Transfection with shABCG2-2 or shABCG2-3 reduced Nanog expression in ES cells (Fig. 2E, G). Immunofluorescence staining confirmed the downregulation of ABCG2 by the shRNA constructs (Fig. 2C–D). As shown in Fig. 2C, GFP (a marker of transfection) colocalized with low levels of ABCG2 staining demonstrating that transfection with shABCG2-3 downregulated ABCG2 expression in these mouse ES cells. Expression of Oct-4 was not altered significantly in shABCG2-3 transfected cells, as there were no GFP-positive cells that colocalized with reduced Oct-4 staining (data not shown). However, expression of Nanog was downregulated in shABCG2-3 transfectants (Fig. 2D). RT-PCR of GFP-sorted transfected cells confirmed this data (Fig. 2E) and utilizing real-time PCR we found that transfection with both shABCG2 construct was sufficient to reduce ABCG2 expression by approximately 70% (Fig. 2F). Nanog expression was reduced by approximately 60% (Fig. 2G). This result was consistent with the results that were obtained through chemical inhibition of ABCG2. Based on these it appears that ABCG2 is regulating the expression of Nanog.

### Self-renewal and Oct-4 is affected in long-term ABCG2 inhibition

**Figure 3:**
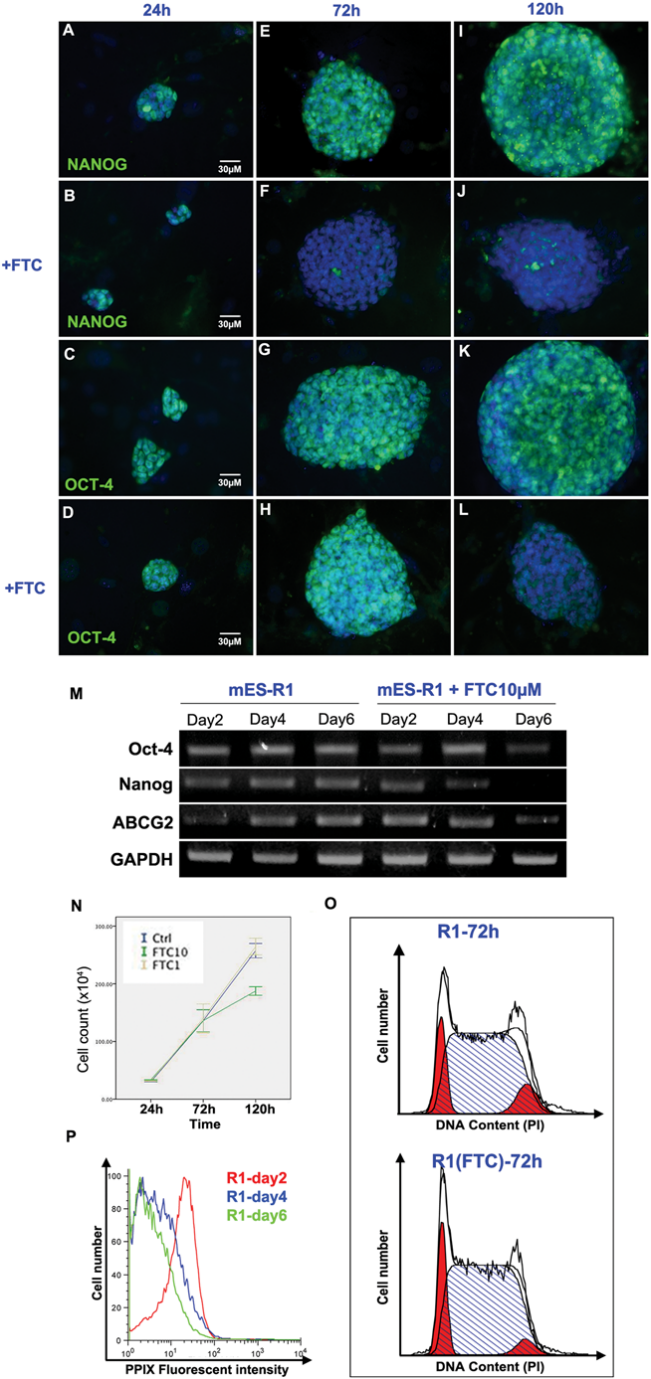
Time course experiments revealed inhibition of ABCG2 for longer period downregulated Nanog and Oct-4 and led to cell cycle arrest at S phase. (A–L) Immunofluorescence staining for Nanog and Oct-4 on untreated and 10 µM FTC-treated mES-R1 cells at 24, 72 and 120 hours after addition of FTC. (M) RT-PCR data for ABCG2, Nanog and Oct-4 in untreated and FTC-treated mES cells at 24, 72 and 120 hours after addition of FTC, with GAPDH as internal control. (N) Growth curves of untreated mES-1 cells and cells treated with 1 µM or 10 µM FTC at 24, 72 and 120 hours after addition of FTC. Error bars represent 1 standard deviation calculated from 3 separate experiments. (O) Cell cycle profile based on PI staining of untreated and 10 µM FTC-treated mES-R1 cells at 72 hours after addition of FTC. Diagram shown represents at least 3 separate experiments. (P) Time course of PPIX expression level in mouse ES cells showing the gradual decrease of PPIX.

To investigate further the effect of ABCG2 inhibition in ES cells we decided to perform a time-course experiment, analyzing the expression of Oct-4 and Nanog at 24, 72 and 120 hours after addition of FTC. Results of time course experiments suggest that after 5 days of FTC treatment, the reduced expression of Nanog possibly led to the subsequent reduction of its downstream target, Oct-4 (Fig. 3A–L, M). Cell growth was also suppressed by FTC treatment after 72 hours of treatment as shown by the growth curve (Fig. 3N). To identify the cause of the reduced growth rate we analyzed the cell cycle profile of untreated and FTC treated cells (Fig. 3O). Here we found the percentage of FTC-treated cells in S phase was higher than that seen in control cells at 72 hours after addition of FTC (70.83%±1.02%vs 64.74%±0.31%). We also observed a decrease in the percentage of cells in G2/M phase in the 10 µM FTC-treated samples compared with control samples (7.25%±0.91% vs 13.36%±2.53%), while the percentages of cells in G1 remained relatively similar between FTC-treated and untreated samples (21.92%±1.19% vs 21.90%±2.85%). Taken together this would indicate that there is an S-phase cell cycle arrest in FTC treated mouse ES cells.

### ABCG2 expression levels during colony expansion is correlated with cytosolic PPIX levels

RT-PCR data from the time course experiment also revealed that ABCG2 expression levels were increased as the colony expands (Fig. 2M). We then wondered if this increase in ABCG2 expression could be correlated with the levels of its substrates-porphyrins. Time course experiments showed that increases in the levels of ABCG2 during colony expansion were followed by a decrease in PPIX levels (Fig. 2N), indicating that ABCG2 might play a role in porphyrin homoeostasis in expanding colonies.

**Figure 4:**
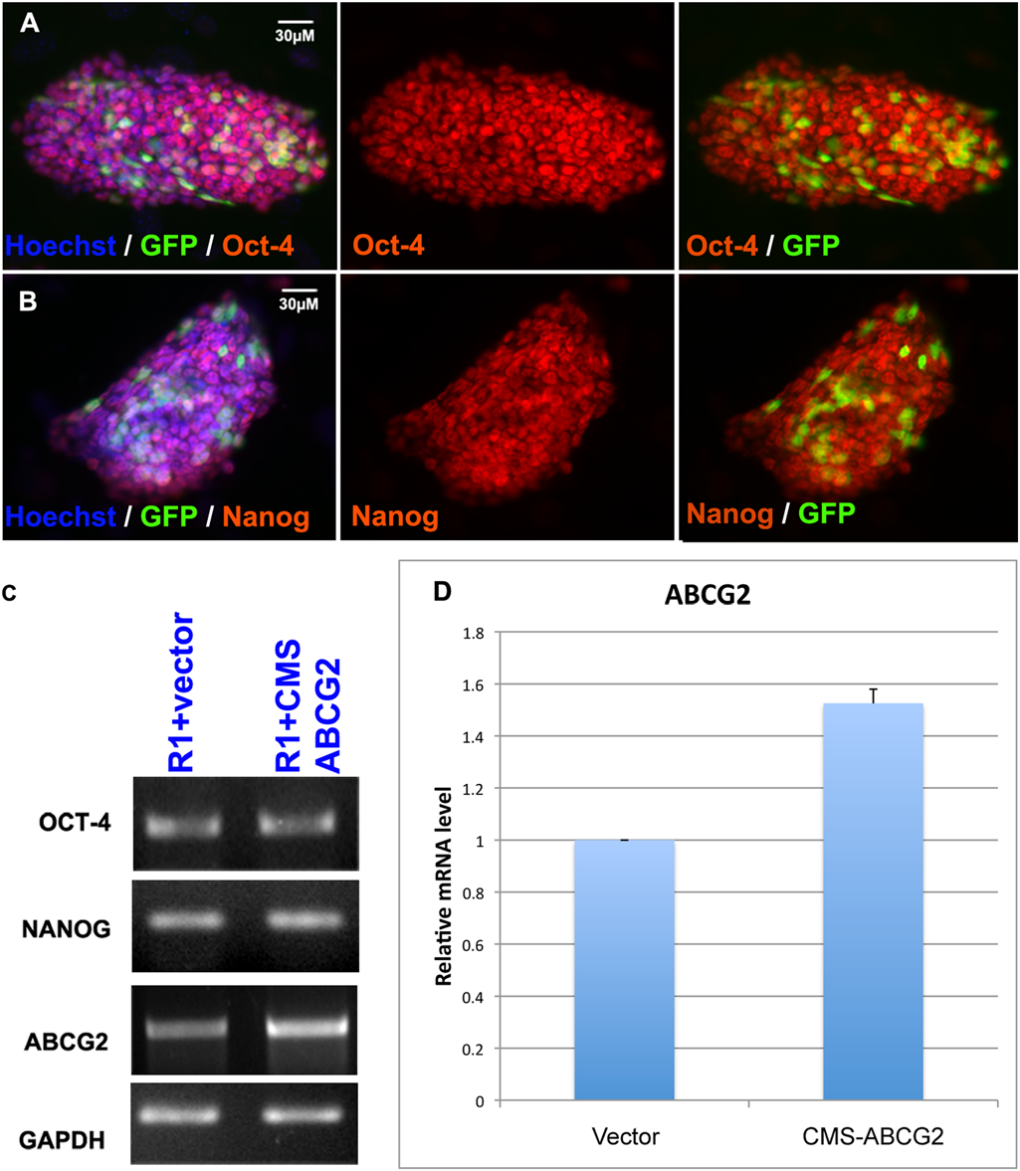
Overexpression of ABCG2 did not alter the level of Nanog and Oct-4. (A, B) Immunofluorescence staining for mES-R1 cells transfected with CMS-ABCG2. The GFP marks transfected cells and red represents Oct-4 (A) and Nanog (B) Immunofluorescence staining at 72 hours after transfection. (C) RT-PCR data for Oct-4, Nanog and ABCG2 for mES-R1 cells transfected with control vector or CMS-ABCG2 with GAPDH as internal control. (D) Real-time PCR showing relative mRNA expression level of ABCG2 in mES-R1 cells transfected with control vector or CMS-ABCG2 normalized to GAPDH. Data was calculated from at least three independent experiments.

**Figure 5:**
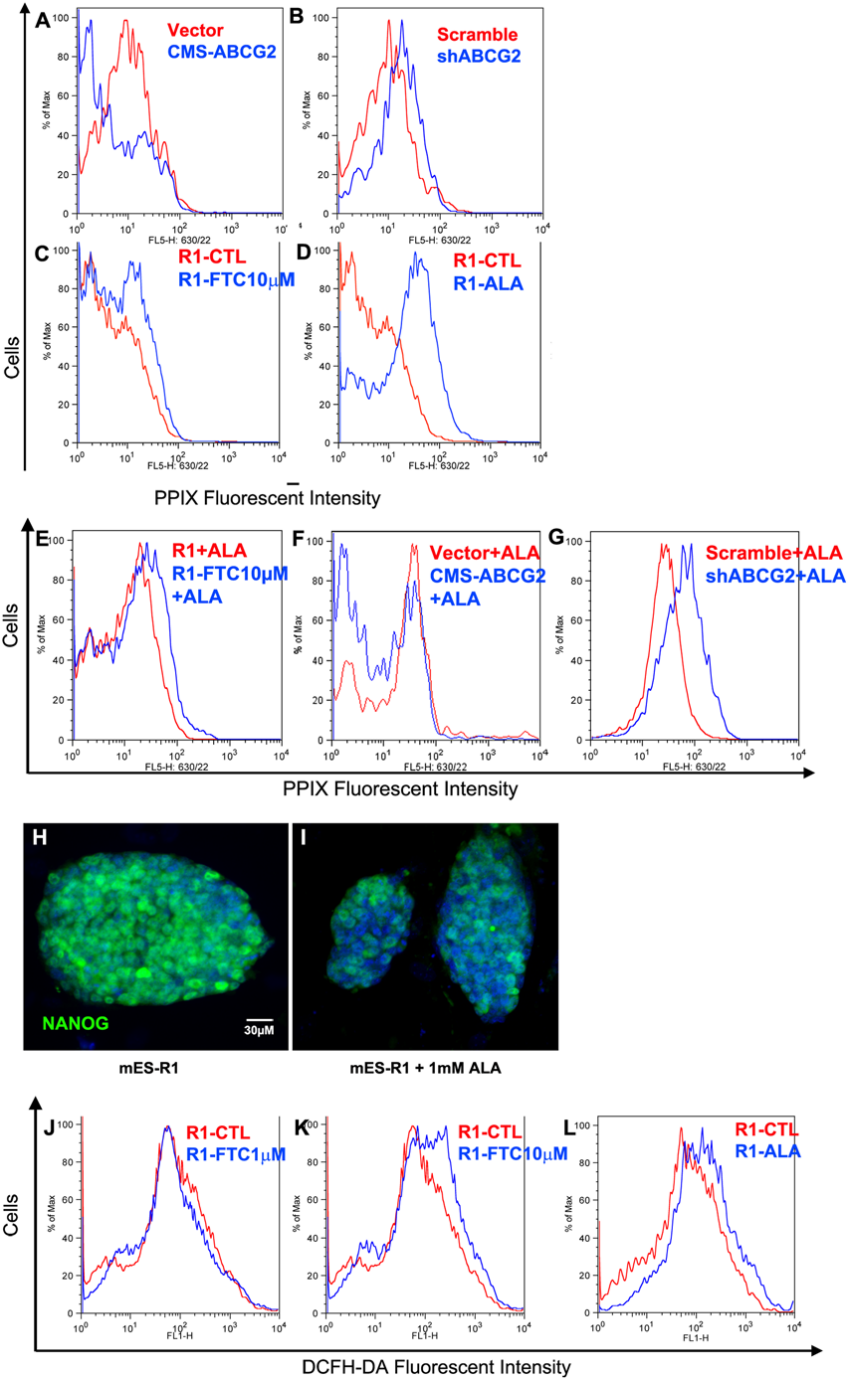
Inhibition of ABCG2 resulted in increases in PPIX and ROS levels. (A–D) PPIX fluorescence measured by flow cytometry for control vector and CMS-ABCG2 (A), scramble and shABCG2 transfected ES cells (B) as well as untreated and 10 µM FTC-treated mES-R1 cells 96 hours after addition of FTC (C). For positive control, mES-R1 cells were treated with 1 mM 5-aminovaleric acid (ALA) for 1 hour to induce production of PPIX (D). Diagram shown represents at least three independent experiments. (E–G) Cytosolic PPIX level measurements after induction with 1 mM ALA for 30 minutes and efflux for 1 hour for Untreated and 10 µM FTC (E) treated mES-R1 control vector and CMS-ABCG2 transfected mES-R1 (F) and scramble and shABCG2 transfected mES-R1(G). (H, I) Immunofluorescence staining showing downregulation of Nanog in untreated (H) as well as 2-hour ALA treated (I) mES cells. (J–L) ROS level measurements by flow cytometry using the ROS sensitive dye DCFH-DA for untreated and 1 µM (J) and 10 µM (K) FTC-treated mES-R1 cells as well as 2-hour treatment with ALA (L). Diagram shown represents at least three independent experiments.

We constructed an overexpression construct by putting a full-length mouse ABCG2 in the mammalian expression vector pCMS-EGFP. We found no difference in the levels of Oct-4 and Nanog in ES cells transfected with this construct (Fig. 4A–C) despite the cellular levels of ABCG2 being increased by approximately 60% (Fig. 4B, C). However, overexpression of ABCG2 decreased the endogenous level of PPIX suggesting ABCG2 might play a role in transportation of cytosolic porphyrins (Fig. 5A).

### ABCG2 inhibition leads to accumulation of PPIX and elevated levels of ROS

In order to determine whether ABCG2 is involved in the transportation of cytosolic porphyrin in ES cells, we measured the level of cellular PPIX in ES cells treated with either 1 mM δ-aminolevulinic acid (ALA) or 10 µM FTC as well as transfection of shABCG2 constructs. ALA is a known precursor of PPIX [20], [28], [29], which indeed induced accumulation of PPIX in ES cells (Fig. 5D). As expected, when ABCG2 was inhibited with either shABCG2 or FTC we see an increase in the level of cellular PPIX (Fig. 5B, C). However, these measurements were done on ES cells that have been grown for 4 days. In order to confirm that accumulation of PPIX in ES cells was directly due to lack of PPIX-transportation by ABCG2, we needed to perform the assay over a shorter period. We decided to do this by inducing high levels of PPIX in ES cells with 1 mM ALA treatment and allowing 1 hour for efflux. Here, we observe that FTC treated as well as shABCG2 inhibited ES cells did show a reduced ability to efflux PPIX (Fig. 5E, G). Conversely, overexpression of ABCG2 in ES cells increased their ability to efflux PPIX (Fig. 5F). These finding suggests that ABCG2 possibly play an important role in maintaining PPIX homeostasis in ES cells.

At this point we decided to see if this accumulation of PPIX was the cause for the phenotype seen in ABCG2 inhibited ES cells. We induced high levels of PPIX in ES cells with 2-hour brief treatment with 1 mM ALA and allowing 2 days for ES cells to adapt. We found that the level of Nanog was significantly reduced indicating accumulation of PPIX by itself without ABCG2 inhibition directly lead to downregulation of Nanog.

### Elevated levels of ROS by excess PPIX activated DNA damage signals

**Figure 6:**
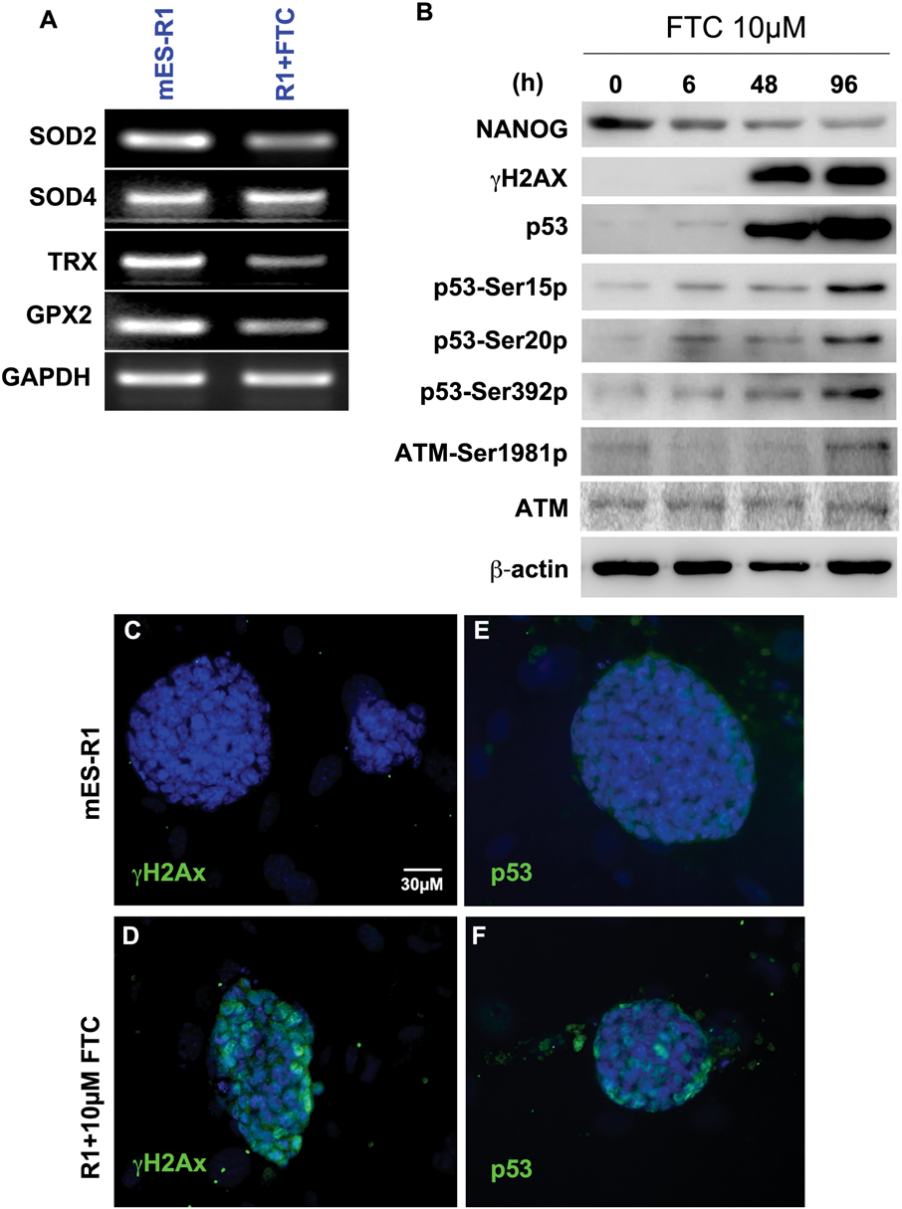
Inhibition of ABCG2 resulted in increases in levels of γH2AX, p53, phosphorylated p53 and phosphylated ATM. (A). RT-PCR data for genes involved in oxidative stress defense: SOD2, SOD4, TRX, and GPX2, with GAPDH as internal control for untreated and 10 µM FTC-treated mES-R1 cells 72 hours after addition of FTC. (B) Western blot of 10 µM FTC-treated mES-J1 cells stained for Nanog, γH2AX, p53 and Phospho-p53 Ser18, 23 and 389 as well as ATM and Phospho-ATM ser1981. Equal amounts of protein were used (40 µg per lane). (C–F) Immunofluorescence staining for γH2AX (C, D) and p53 (E, F) in untreated and 10 µM FTC treated mES-R1 cells taken at 72 hours after addition of FTC.

It has been shown that ROS can be generated by oxidation of the porphyrin precursor ALA [30] or by the fenton reaction involving PPIX [16]. Since both ALA and FTC treatment increased the level of PPIX, it would follow that they would lead to increased levels of reactive oxygen species (ROS). Indeed this was what we observed in 10 µM FTC treated, and ALA treated mES cells (Fig. 5J–L). RT-PCR analysis further revealed that superoxide dismutase 2 (SOD2), thioredoxin (TRX), and glutathione peroxidase 2 (GPX2) levels were reduced after FTC treatment, indicating that the endogenous expression of genes involved in oxidative stress defense was not sufficient to remove excessive ROS (Fig. 6A).

Since ROS are a major source of DNA damage, we hypothesised that elevated ROS in ES cells may trigger DNA-damage signals. Time course experiments confirmed that suppression of the ABCG2 transporter with FTC did in fact increase the level of phosphorylated H2AX (γH2AX) (Fig. 6B, D). This suggests that DNA double strand breaks (DSBs) had occurred, which may explain the cell cycle arrest at S phase we observed in (Fig. 3O). Furthermore, ES cells with oxidative damage demonstrated a reduction of Nanog accompanied by phosphorylation of ataxia telangiectasia mutated (ATM) at Ser 1981. This in turn led to an increase in the level of p53 and p53 phosphorylation at ser18, ser23, and ser389 (Fig. 6B) in response to DNA damage signals [31]–[33].

### Downregulation of Nanog in ABCG2 inhibited ES cells is a p53 dependent pathway

**Figure 7:**
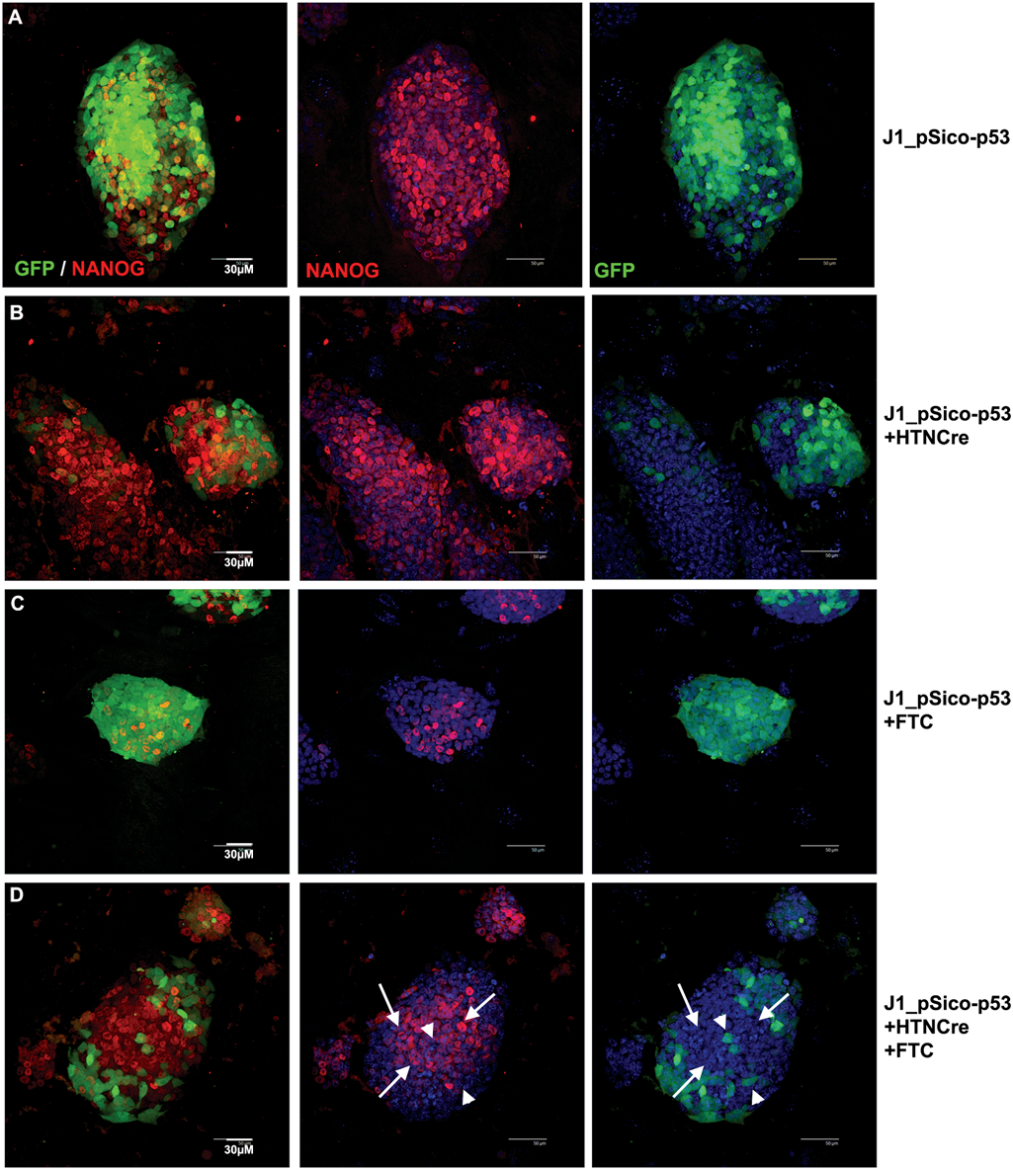
Knock-down of p53 prevented downregulation of Nanog in ABCG2-inhibited ES cells. (A–D) Immunofluorescence staining for Nanog on untreated (A, B) and 10 µM FTC-treated (C, D) J1_pSico-p53 cells with (B, D) or without (A, C) the addition of HTNCre to activate inhibition of p53. White arrows indicate GFP negative cells with high Nanog where p53 inhibition has rescued Nanog expression. Arrowheads indicate GFP positive cells with low or no Nanog.

Previous studies have shown that p53 binds to the promoter of Nanog and suppresses Nanog expression after UV-induced DNA damage. We observed nuclear localization of p53 (Fig. 6F), therefore we want to determine whether the downregulation of Nanog in ABCG2-inhibited ES cells was directly mediated by p53, we generated a GFP-labelled ES cell line containing a Cre-lox-regulated p53 RNA interference construct (named J1_pSico-P53, Fig. 7A) [34]. As shown in Fig. 7B, HTNCre-mediated excision led to recombination, with loss of GFP fluorescence within 2 days accompanied by expression of p53-shRNA and decreases in the level of p53 (data not shown), which did not alter the level of Nanog significantly. FTC treatment dramatically suppressed Nanog expression in J1_pSico-P53 ES cells (Fig. 7C). In contrast, the combined treatment with HTNCre and FTC showed that p53-shRNA-expressing cells (GFP-negative cells, arrows in Fig. 4E) maintained the expression of Nanog that is higher compared to adjacent GFP-positive cells, which did not express p53-shRNA (arrow heads in Fig. 6E). These results confirm that p53 mediates the downregulation of Nanog in ABCG2-inhibited ES cells.

## Discussion

**Figure 8:**
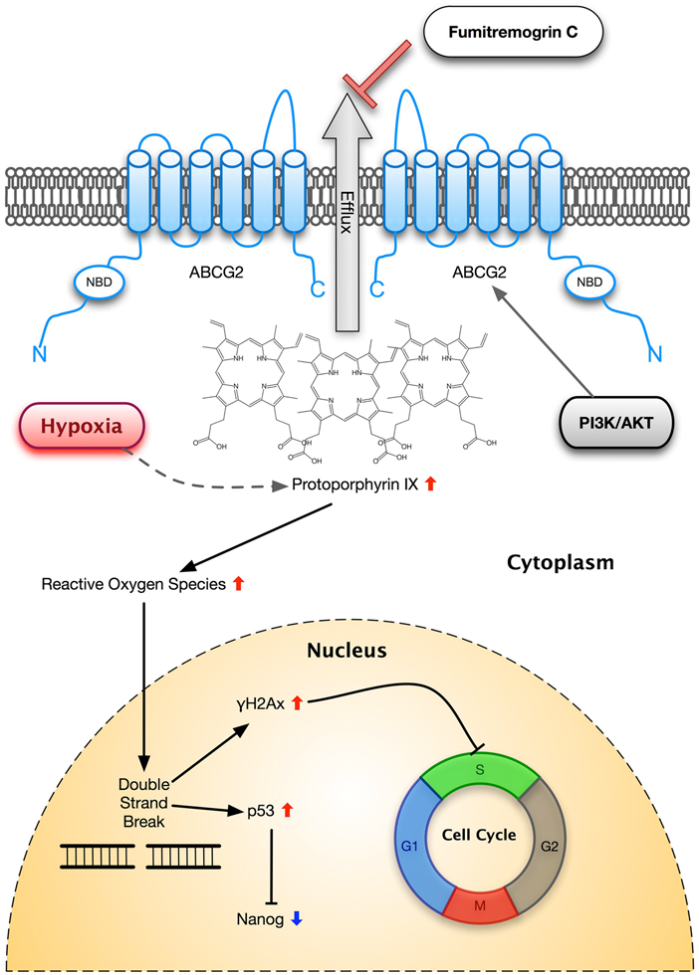
Summary of proposed role of ABCG2 in mouse ES cells. Diagram illustrating how inhibition of ABCG2 could lead to accumulation of PPIX and elevated levels of ROS, which in turn caused DNA damage and eventually activating p53 and γH2AX, Activation of p53 lead to downregulation of Nanog whilst γH2AX causes cell cycle arrest. Also depicted in the diagram is possible involvement of PI3K/AKT a well as hypoxia in this pathway.

The ABCG2 multidrug-transporter is widely expressed in a variety of tissue stem cells and cancer stem cells, suggesting that it plays an important role in self-renewal maintenance. We have identified an endogenous role of ABCG2 in maintaining the self-renewal of ES cells during self-renewal cycles. We demonstrate that disruption of ABCG2 function in ES cells can lead to accumulation of PPIX over time. This idea is supported by the fact that porphyrins accumulate in tissues of the BCRP knockout mouse [23]. One of the consequences of the accumulation of PPIX is elevated ROS level in ES cells leading to an increased potential for DNA damage (Fig. 8).

Indeed, we found an increase in both γH2AX and pATM levels in ABCG2 inhibited ES cells, indicating DNA damage. Phosphorylation of ATM in response to ROS induced DNA damage leads to the activation of p53 [4], [35]. It has been shown that ES cells do not have G1 checkpoints and only undergo p53-independent apoptosis after certain types of physiological DNA damage, possibly due to p53 being sequestered in the cytoplasm unless SIRT1 is inhibited [36], [37]. Hence the cell cycle arrest we observed is likely caused by γH2AX, preventing proliferation of DNA damaged ES cells. Furthermore, we did not observe significant increases of apoptosis in ABCG2-inhibited ES cells. In contrast, we obtained a dramatic upregulation in the nuclear level of p53, which was able to suppress Nanog expression in ABCG2-inhibited ES cells. The findings probably resulted from direct binding of PPIX to p53, which disrupts the interaction between p53 protein and its negative regulator such as HDM2 [38]. This represents an alternative pathway for maintaining genetic stability in ES cells [39]. Consistent with this notion, our current work has confirmed that p53 can suppress Nanog expression in response to accumulated levels of PPIX and ROS and then trigger the differentiation of “damaged” ES cells into nestin-expressing cells, which effectively removes the damaged cells from the self-renewal pool.

**Figure 9:**
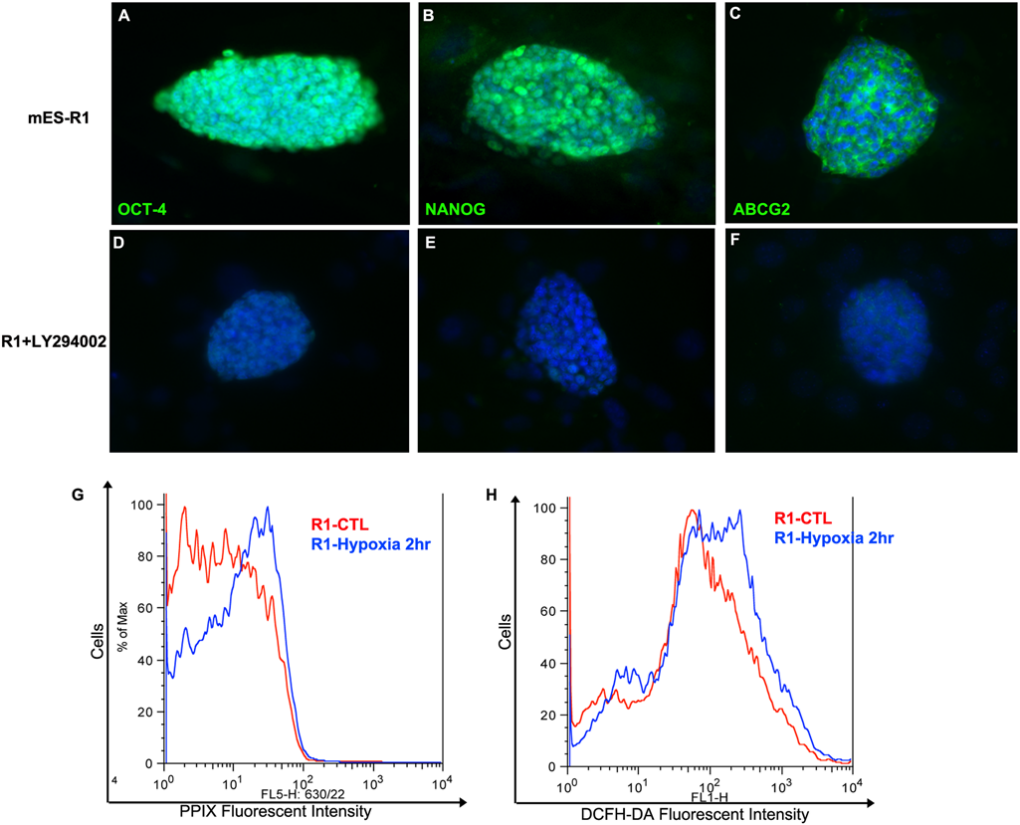
ABCG2 possibly play as a downstream mediator of PI3K/AKT in the self-renewal of ES cells. (A–F) Immunofluorescence staining for ABCG2, Oct-4 and Nanog for untreated and 10 µM LY294002 treated mES-R1 taken at 72 hours after addition of LY294002. Here we can see that ABCG2 was downregulated when PI3K was inhibited (G) PPIX fluorescence measures showing an increase in 2 hour-hypoxia treated mES-R1 cells compared to control cells. (H) DCFH-DA staining showing increase in the ROS levels in 2 hour-hypoxia treated mES-R1 compared to control cells.

Recent findings have indicated that the PI3K/Akt signaling pathway regulates the surface expression of ABCG2 [40] and may be involved in regulating the pluripotency of ES cells. Inhibition of PI3K with the PI3K inhibitor LY294002 in mouse ES cells reduced self-renewal growth and downregulated Nanog expression [41]. Likewise, we found that treatment of mouse ES cells with the PI3K inhibitor-LY294002 led to inhibition of the side-population phenotype (data not shown) as well as downregulation of Oct-4 and Nanog expression (Fig. 9A, B, D, E), which is consistent with previously published results. Moreover, we discovered that the expression of ABCG2 was also downregulated in these LY294002-treated ES cells (Fig. 9C, F), suggesting that ABCG2 acts downstream of PI3K.

ES cells have a high proliferative rate, allowing for rapid expansion of ES colonies, which we speculate may reduce oxygen availability and resulted in alteration of heme synthesis and metabolism in some ES cells. Indeed we found a brief hypoxic treatment (10% O_2_ for 2 hours) led an increase in both the cytosolic level of PPIX and ROS (Fig. 8G, H) in ES cells. In accordance with this, we speculate that the variance seen in the expression of ABCG2 in different mES cells could be correlated with their oxygen availability and proliferation rate. Indeed, from the four different mES cells tested B6 is known to have a slower proliferation rate compared to R1 and J1, which might explain the lower ABCG2 expression seen in B6 compared to R1 and J1. Furthermore, ABCG2 have also been shown to enhance cell survival under hypoxia [15] and contain at least one functional hypoxia response element in its promoter [12], which would suggest an involvement of hypoxia-inducible factors in the regulation of ABCG2. This hypothesis is supported by a recent study, which discovered transactivation of ABCG2 by hypoxia-inducible factor 2 alpha in cardiac SP cells [42]. Also, considering that PPIX levels are increased during hypoxia it makes it very likely that hypoxia-inducible factors might be involved in regulating ABCG2 levels with respect to regulate PPIX homeostasis.

In conclusion, we have demonstrated here for the first time that the ABCG2 transporter plays a role in preventing porphyrin accumulation in ES cells and excessive production of ROS from heme metabolism during colony expansion. When this function is inhibited, excess PPIX would lead to trigger DNA damage signals such as pATM, pp53 and γH2AX, which function as downstream checkpoints to ensure that the genetic integrity of ES cells is maintained and/or to eliminate ES cells with damaged DNA from the self-renewal pool.

## Materials and Methods

### ES cell culture

Wild type ES cell lines R1, J1, D3 and B6 were cultured on mitotically inactivated mouse embryonic feeder (MEF) cells in Dulbecco's Modified Eagle medium (DMEM, Hyclone) with 10% ES quality foetal bovine serum (FBS; Hyclone), 1 mM sodium pyruvate (Gibco), Penicillin (100units/ml)/Streptomycin (100 µg/ml) (Gibco), 5.5×10^−2^ mM β-mercaptoethanol, (Calbiochem) and 10^3^ U/ml LIF (Chemicon). Embryoid bodies (EBs) were formed by seeding the ES cells at densities of 500–2000 cells/cm^2^ on uncoated plates.

### Inhibitor Treatment

The chemical inhibitors LY294002 (Sigma) and Fumitremogrin C (Calbiochem) were added to the ES growth media 24 hours after the ES cells were seeded on MEF cells (day 1). The media containing inhibitors was changed daily to ensure activity. Cells were treated for various time periods before being subjected to immunostaining or RT-PCR analysis.

### PPIX and ROS measurements

mES cells were harvested using Collagenase IV (Sigma) for 5 minutes at 37°C and washing once with staining buffer (HBSS; Gibco)+2% Serum (Gibco) and 1 mM HEPES (Gibco) before protoporphyrin IX and ROS concentrations were assessed using a Vantage flow cytometer (BD Biosciences). Endogenous PPIX levels were measured by using UV laser excitation and measuring the emission using a 635 nm filter with Vantage flow cytometry (BD Biosciences). To determine the ROS levels, mES cells were incubated with 10 µM of freshly prepared DCFH-DA dye in HBSS (Gibco) for 30 minutes at 37°C, and were washed twice with staining buffer before measurement using Callibur flow cytometry (BD Biosciences) with an FITC emission filter.

### PPIX efflux

To determine the ability of cells to efflux PPIX, mES cells were incubated for 30 minutes at 37°C in ES media with 1 mM ALA followed by a further 1 hour incubation in ES media with or without inhibitors before measurement of PPIX levels by using UV laser excitation and measuring the emission using a 635 nm filter with Vantage flow cytometry (BD Biosciences).

### Plasmids construction and transient transfection

Two different shRNAs, shABCG2-2 and shABCG2-3, were designed to target within the ABCG2 CDS region (see Table 1 for sequence), and were cloned into the pDsiPHER-GFP plasmid (Molecula). A scrambled version of shABCG2-2 was also made for control. The ABCG2 overexpression vector was constructed by subcloning a full-length mouse ABCG2 cDNA obtained from Invitrogen (Clone ID 30064535) into the mammalian expression vector pCMS-EGFP (Clontech). Once constructed, the plasmids were transfected into the ES cells using Fugene HD transfection reagent (Roche). The ES cells were then cultured for another 48-72 hours before immunostaining or sorting using BD FACS Aria cell sorter for GFP to use for RT-PCR.

**Table 1:** shABCG2 and scramble control sequence.

Target	Sense
shABCG2-2	GATCC**GGATTAACACAATCATTAA**TTCAAGAGA**TTAATGATTGTGTTAATCC**TTTTTTA
shABCG2-3	GATCC**GCCCTGGCTTGTATGATTA**TTCAAGAGA**TAATCATACAAGCCAGGGC**TTTTTTA
scramble	GATCC**GGTCGAATTTGCGAACCAA**TTCAAGAGA**TTGGTTCGCAAATTCGACC**TTTTTTA

### Establishment of J1_pSico-p53 ES cell lines

J1 mouse ES cells were seeded on 6 cm tissue culture plates with mitomycin C inactivated MEF. On day 1, cells were infected with lentivirus-pSico_p53 and polybrene 8 µg/ml and were left overnight. Cells were then washed with PBS, incubated in ES media, and cultured for an additional 3 days. To select for lentivirus infected J1 mES cells, the cells were detached by collagenase IV then trypsinized by 0.1% trypsin and sorted for GFP signal in BD FACS Aria cell sorter. The GFP positive J1-pSico_p53 cells were grown on mitomycin C inactivated MEF for further use.

In order to produce pSico_p53 lentivirus, the day prior to transfection, confluent 10-cm plates of 293T cells were seeded at 2.4×10^6^ cells/plate. The expression plasmid pSico_p53 was obtained from Addgene (plasmid 12089) [43]. For each 10-cm dish, 7.5 µg expression vector, 6.75 µg pCMV-Δ8.91 packaging plasmid, and 0.75 µg pMD.G envelope were transfected using Genejuice transfection reagent (Novagen). The day after transfection, the media was replaced with fresh media. Culture supernatant was subsequently harvested at 48, 72 and 96 hours post-transfection. Virus-containing supernatant was filtered through 0.45 µm pore filters and stored at 4°C. Virus was further concentrated by ultracentrifugation for 2.5 hours at 26 000 rpm in a Beckman SW 28.1 rotor (Beckman Coulter), and the resulting virus pellet was resuspended in PBS (pH 7.4) containing 1% BSA at 4°C overnight before being aliquoted and stored at −80°C.

### HTNcre Cre treatment of J1_pSico-p53

Sorted J1_pSico-p53 cells were cultured in HTNCre containing medium (5 mM) overnight, were washed twice with PBS, and then treated with FTC 10 µM for 3–4 days before immunostaining. HTNCre protein was produced as previously described [44].

### Immunofluorescence Staining

Cells were fixed in 4% paraformaldehyde for 30 minutes at room temperature and permeabilized at room temperature in 0.1% Triton X-100 for 30 minutes. After blocking with 2% Roche blocking reagent, the cells were incubated with primary antibody overnight at 4°C and with secondary antibody for 2 hours at room temperature. The primary antibodies were used at the following dilutions: Oct4 (1∶200, Santa Cruz); Nanog (1∶150, Reprocell); Carbonyl (1∶62, Chemicon Oxyblot kit); Nestin (1∶300, Chemicon); ABCG2 (1∶75, Chemicon); p53 (1∶300, Santa Cruz); γH2AX (1∶200, Upstate). The secondary antibodies were used at the following dilutions: goat anti-rabbit fluorescein (1∶300, Chemicon), goat anti-mouse fluorescein (1∶300, Vectorlabs), goat anti-rabbit Texas Red (1∶300, Vectorlabs) and goat antimouse Texas Red (1∶300, Vectorlabs). Images of the immunostaining were obtained using a SPOT camera mounted on a Zeiss Axioplan 2 microscope.

### RT-PCR and real-time PCR

Cells used for RT-PCR experiments were washed once with PBS before RNA extraction with Trireagent (Ambion) per manufacturer's instructions. 5 µg of the purified RNA was then subjected to reverse transcription using Superscript III (Invitrogen) per manufacturer's instructions to obtain cDNAs for PCR. Real-time PCR was performed using Lightcycler 2.0 (Roche) with the SYBR green Taqman master kit (Roche) per manufacturer's instructions. Details of the sequence of primers used in PCR can be found in Table 2.

**Table 2:** List of Primers used in RT-PCR.

Target	Forward Primer	Reverse Primer
Nanog	AAAGGATGAAGTGCAAGCGGTGG	CTGGCTTTGCCCTGACTTTAAGC
Oct-4	AGGAAGCCGACAACAATGAG	CTGATTGGCGATGTGAGTGA
ABCG2	CCATAGCCACAGGCCAAAGT	GGGCCACATGATTCTTCCAC
GAPDH	AAGGTCGGTGTGAACGGATT	TGGTGGTGCAGGATGCATTG
SOD2	AGGTCGCTTACAGATTGCTG	GTGTCGATCGTTCTTCACT
SOD4	TATCGATGAGGGGGAAGATG	CTCGCTCCTCCCAGATAGTG
TRX	GTCTCTTTAGAAAAGTGTGA	ATTGCAGCTGCAAATCCCTG
GPX2	ATTGCCAAGTCGTTCTACGA	GTAGGACAGAAACGGATGGA

### SDS-PAGE and Immunoblot Analysis

Samples were separated by SDS-PAGE (10%) and subsequently transferred to PVDF Immobilon-P membrane (Millipore). Samples were incubated in blocking buffer (0.1% Tween 20, 5% nonfat milk powder in TBS) for 1 hour at room temperature. Afterwards, the membrane was incubated with primary antibody in blocking buffer overnight at 4°C before being washed twice with TBST (0.1% Tween in TBS) and incubated with the appropriate secondary antibody in blocking buffer for 1 hour at room temperature. The blot was developed using the Western Chemiluminescent HRP substrate (Immobilon). The primary antibodies were used at the following dilutions: Nanog (1∶400, Reprocell); p53 (1∶400, Cell Signaling), p53-Ser15p (1∶400, Cell Signalling), p53-Ser20p (1∶400, Cell Signaling), p53-Ser392p (1∶400, Cell Signaling), ATM (1∶200, Cell Signaling), pATM-Ser1981 (1∶250, Cell Signaling); γH2AX (1∶400, Upstate). The secondary antibodies used were anti-rabbit HRP (1∶1000, Santa Cruz) or anti-mouse HRP (1∶1000, Santa Cruz).

### Cell Cycle Profile

Mouse ES cells were harvested with Collagenase IV (Sigma) and washed once with PBS before being fixed with dropwise addition of 1 ml ice cold 70% EtOH and incubated overnight at −20°C. The following day, the cells were washed once with PBS and stained with cell cycle staining buffer (1% Triton X-100 (Sigma), 0.2 mg/ml RNAse A (Promega) and 0.02 mg/ml propidium iodide in PBS) for 30 minutes at room temperature. The cells were then washed twice with PBS before propidium iodide fluorescence was measured using Callibur flow cytometry (BD Biosciences). Thepercentages of cells in each phase of the cell cycle were calculated using the Modifit LT software package.
